# Metabolic Implications of Elevated Neutrophil Extracellular Traps in Polycystic Ovary Syndrome: A Focus on Hepatic Glycolysis

**DOI:** 10.3390/biom15040572

**Published:** 2025-04-12

**Authors:** Siyu Lin, Yushan Li, Wei Liu, Yanzhi Du, Tao Tao

**Affiliations:** 1 Department of Endocrinology and Metabolism, Ren Ji Hospital, Shanghai Jiao Tong University School of Medicine, Shanghai 200127, China; linheihei6@sjtu.edu.cn (S.L.);; 2 Center for Reproductive Medicine, Renji Hospital, Shanghai Jiao Tong University School of Medicine, Shanghai 200135, China; 3 Shanghai Key Laboratory for Assisted Reproduction and Reproductive Genetics, Shanghai 200135, China

**Keywords:** polycystic ovary syndrome (PCOS), neutrophil extracellular traps (NETs), insulin resistance, untargeted metabolomics

## Abstract

**Background:** Polycystic ovary syndrome (PCOS) is a leading cause of infertility but also a metabolic disorder, frequently associated with obesity, insulin resistance, and diabetes. However, its etiology remains inadequately understood. Recent studies have increasingly implicated neutrophil extracellular traps (NETs) in the pathogenesis of metabolic diseases. **Methods:** Serum and follicular fluid samples were collected from patients with PCOS and control populations to assess NETs levels. The effects of NETs were investigated using DNase I to reduce NETs in dehydroepiandrosterone sulfate (DHEAS)-induced PCOS rats. Metabolic differences were further analyzed by untargeted metabolomics, and in vitro studies were conducted using primary bone marrow-derived neutrophils and normal mouse liver cell lines. **Results:** Markers of NETs in both the serum and follicular fluid of patients with PCOS were significantly higher than those in the control group. PCOS rats treated with DNase I exhibited significant improvements in glucose metabolism. Untargeted metabolomics analysis of liver tissue from these rats revealed alterations in the glycolysis pathway. Subsequent in vitro experiments demonstrated that treatment with NETs-conditioned medium (NETs CM) led to reduced insulin sensitivity, glucose uptake, and glucose utilization in liver cells, accompanied by varying degrees of decline in the transcription, translation, and function of glycolysis pathway proteins. **Conclusions:** NETs may be involved in the regulation of insulin resistance pathogenesis in PCOS by downregulating glycolytic pathways in the liver. Our study offers a novel strategy for insulin resistance intervention in PCOS.

## 1. Introduction

Polycystic ovary syndrome (PCOS) is a prevalent and multifaceted endocrine and metabolic disorder affecting women of reproductive age, characterized by neuroendocrine, metabolic, reproductive, and psychological abnormalities. The syndrome is typically marked by chronic anovulation and hyperandrogenism, presenting clinically as irregular menstruation, infertility, hirsutism, and acne, and is frequently accompanied by metabolic disorders such as obesity and insulin resistance [1]. According to the Global Burden of Disease 2019, (GBD 2019), the global age-standardized point prevalence and annual incidence of PCOS were 1677.8 and 59.8 per 100,000 individuals, respectively, reflecting increases of 30.4% and 29.5% since 1990, thereby imposing a substantial societal burden [2]. Regarding the prevalence of PCOS in China, a study reported that the prevalence among women aged 20–49 years in 2020 was 7.8% [3]. The etiology of PCOS remains elusive and may involve genetic and epigenetic influences, maternal pregnancy environment, lifestyle, substance exposure, and psychological anxiety [4,5,6]. Furthermore, women with PCOS exhibit a variety of risk factors for cardiovascular disease and diabetes, underscoring the critical importance of investigating the etiology and pathogenesis of PCOS.

Emerging evidence suggests that chronic low-grade inflammation is also present in PCOS, characterized by elevated levels of C-reactive protein (CRP), increased white blood cell counts, and heightened pro-inflammatory cytokines [7]. Elevated androgen levels are known to stimulate chronic low-grade inflammation in the ovaries, activating the NLRP3 inflammasome, which subsequently induces pyroptosis of ovarian granulosa cells, follicular dysfunction, and fibrosis of ovarian stromal cells [8]. Furthermore, exposure to an imbalanced lipid profile may exacerbate ovarian inflammation [9].

As research into immune–endocrine and immune–metabolic interactions advances, investigations into the role of immune cells in the pathogenesis and progression of PCOS have intensified. It has been documented that the white blood cell count in the peripheral blood of patients with PCOS and hyperinsulinemia increases with the production of hyperandrogens, primarily in macrophages and neutrophils [10].

Neutrophils, as integral components of the innate immune response, serve as crucial effector cells that play a significant role in immune surveillance and the detection of microbial infections. In inflammatory conditions, neutrophils exhibit various activation states, including phagocytosis, degranulation, and the formation of neutrophil extracellular traps (NETs) [11]. NETs are extensive extracellular reticular structures composed of cytosolic and granular proteins, assembled on scaffolds of depolymerized chromatin. Since the initial discovery of NETs in 1996 [12], Brinkmann et al. elucidated the detailed process of NETs formation, subsequently termed NETosis, in 2004 [13]. While neutrophils were primarily associated with the initiation and progression of acute inflammation, recent findings have highlighted the pivotal role of NETs in chronic inflammation associated with metabolic diseases [14,15,16]. In the context of metabolic diseases, research has indicated that NETs are significantly elevated in the plasma of obese patients compared to control groups [17]. Furthermore, stimulation of neutrophils in obese individuals has been shown to increase superoxide production to varying degrees, suggesting an alteration in neutrophil function in these patients [18]. Similarly, neutrophil reactivity is heightened in individuals with diabetes; upon stimulation, diabetic patients’ neutrophils are more prone to undergo NETosis than those of healthy controls [19]. Both in vivo and in vitro studies have demonstrated that hyperglycemia facilitates the release of NETs [20]. Moreover, NETs are implicated in the progression of nonalcoholic fatty liver disease (NAFLD) and are closely associated with steatosis, ballooning, lobular inflammation, portal vein inflammation, and disease staging [21]. This evidence underscores the role of NETs in the progression of metabolic diseases.

In summary, NETs are integral to the pathophysiology of metabolic diseases and hold significant clinical relevance. The conditions associated with PCOS, such as hyperglycemia, obesity, and chronic low-grade inflammation, are hypothesized to facilitate NETs production. Nevertheless, the precise levels of NETs in individuals with PCOS have yet to be documented. This study aims to address this gap by collecting and analyzing clinical serum and follicular fluid samples from both patients with PCOS and a control group. The investigation further examines the role of NETs in the pathogenesis of PCOS through animal and cellular experiments, thereby contributing to the understanding of the etiology of chronic inflammation in PCOS and identifying potential therapeutic targets.

## 2. Materials and Methods

### 2.1. Sample Collection from Patients

A total of 57 individuals with PCOS and 38 age- and body mass index (BMI)-matched control women were recruited from the Department of Endocrinology and Reproductive Center, Renji Hospital, Shanghai Jiao Tong University. The diagnosis of PCOS was established according to the 2003 Rotterdam Criteria. The inclusion criteria required participants to be between 20 and 38 years old, with a BMI exceeding 18. No statistically significant differences in age or BMI were observed between the PCOS and control groups in the two cohorts from which serum and follicular fluid samples were collected, respectively.

Exclusion criteria for all participants were as follows: (1) diagnosis of diabetes, (2) severe abnormalities in liver function tests, and (3) regular use of medications known to affect glucose–lipid metabolism or hormone levels within the past month. The same exclusion criteria were applied to the control subjects.

Serum samples were obtained from 40 PCOS patients and 19 controls at the time of initial diagnosis, prior to any treatment (Table 1). These samples were centrifuged at 4 °C, 3000 rpm for 10 min after standing at room temperature for 2 h to isolate the supernatant. Follicular fluid samples were collected from 17 PCOS patients and 19 controls during the oocyte retrieval step of in vitro fertilization (IVF) treatment. The control patients in this study did not have PCOS and underwent IVF treatment due to other indications, such as male partner infertility or uterine and cervical anomalies (Table 2). Ovarian stimulation and oocyte retrieval were performed in accordance with standard IVF protocols. Follicular fluid samples were collected during oocyte retrieval and centrifuged at 2000 rpm for 10 min at 4 °C to obtain the supernatant. Biochemical index testing for all participants was conducted by the Laboratory Department of Renji Hospital.

### 2.2. NETs Quantification

#### 2.2.1. dsDNA

Double-stranded DNA (dsDNA) levels in serum and follicular fluid were quantified using the Quant-iT PicoGreen DNA Quantification Kit (Invitrogen, Waltham, Massachusetts, USA, P11496) according to the manufacturer’s instructions. Briefly, pure dsDNA standards and samples were incubated with 100 μL of Quant-iT PicoGreen Reagent for 5 min. The fluorescence signal was measured using a microplate reader set to 485 nm (excitation) and 538 nm (emission).

#### 2.2.2. NE

Neutrophil elastase (NE) levels in serum were measured using the Human Neutrophil Elastase ELISA Kit (Abcam, Cambridge, UK, ab270204), following the manufacturer’s protocol. Samples and standards were added to the assay wells, antibodies were introduced, unbound materials were removed through washing, and the signal intensity of the bound material was measured at 450 nm using an enzyme-linked immunosorbent assay reader.

### 2.3. Animals

#### 2.3.1. Model Construction

Three-week-old female Sprague-Dawley (SD) rats were purchased from Beijing Charles River Laboratories. Following a one-week acclimatization period, the rats were maintained under a 12 h light/12 h dark cycle in an environment with controlled temperature and humidity. Body weights were measured and recorded every three days, commencing on the first day of the experimental procedure.

Four-week-old rats were randomly allocated to one of four groups: the control group (Ctrl, *n* = 9), the polycystic ovary syndrome (PCOS, *n* = 8) group, the treatment group (PDNaseI, *n* = 9), or the DNaseI group (DNaseI, *n* = 5), with initial weight serving as the basis for matching. For each rat in the PCOS group, dehydroepiandrosterone sulfate (DHEAs) (Shanghai Yaxing, Shanghai, China) was administrated subcutaneously at a dose of 60 mg/kg/day for 24 days. For each rat in the PDNaseI group, DNase I (0.1 mg/kg) (Roche, 10104159001) was administrated through the tail vein concomitantly with the daily subcutaneous injection of DHEAs for 24 days [22]. Additionally, an equivalent volume of normal saline was injected into the tail vein of each rat in the PCOS group. In the DNaseI group, DNase I (0.1 mg/kg) was administered daily with 200 µL of PBS via subcutaneous injection. For each rat in the control group, 200 µL of PBS was injected subcutaneously each day, with an equivalent volume of normal saline administrated into the tail vein. After the 24-day trial, the rats were euthanized at about 8 weeks of age.

#### 2.3.2. Estrous Cycle Analysis

The estrous cycle stage was determined through microscopic analysis of the predominant cell types in vaginal smears. Vaginal cells were collected by saline lavage during the last 8 consecutive days of the 24-day trial, and daily samples were examined under a light microscope. The proestrus stage was characterized by predominantly nucleated cells, the estrus stage by cornified squamous epithelial cells, the metestrus stage by a mixture of cornified cells and leukocytes, and the diestrus stage by a predominance of leukocytes.

#### 2.3.3. IPGTT

The intraperitoneal glucose tolerance test (IPGTT) was conducted on the 20th day of modeling. After fasting for 17 h, rats were injected intraperitoneally with a 50% glucose solution (2 g/kg body weight), and blood glucose levels were monitored from tail bleeds at 0, 15, 30, 60, 90, and 120 min using a glucometer monitor (Roche).

#### 2.3.4. ITT

The intraperitoneal insulin tolerance test (ITT) was performed on the 24th day of modeling (4 days after IPGTT). After fasting for 4 h (from 10:00 a.m. to 2:00 p.m.), rats were injected intraperitoneally with regular insulin (1 U/kg body weight), and glucose levels were measured at 0, 30, 60, 90, and 120 min using a glucometer.

#### 2.3.5. Measurement of Hormone Levels

Blood samples of rats were collected from the abdominal aorta and centrifuged at 3000–4000 rpm for 10 min at 4°C to obtain the supernatant. Serum levels of dsDNA (Invitrogen™, Waltham, MA, USA, P11496), myeloperoxidase-DNA (MPO-DNA) (MEIMIAN, Jiangsu, China, MM-71053R2), testosterone (T) (Demeditec, Kiel, Germany, DEV9911), luteinizing hormone (LH) (Sangon Biotech, Shanghai, China, D731015), follicle-stimulating hormone (FSH) (CUSABIO, Houston, TX, USA, CSB-E06869r), and insulin (MULTI SCIENCES, Hangzhou, China, EK3220) were measured following the manufacturers’ instructions. The homeostasis model assessment of insulin resistance (HOMA-IR) was calculated using the formula: HOMA-IR = glucose × insulin/22.5.

### 2.4. Immunostaining

Liver tissues were fixed with 4% paraformaldehyde and embedded in paraffin for immunofluorescence (IF) analysis. Anti-myeloperoxidase (MPO) (22225-1-AP, Proteintech, 1:200) and anti-citrullinated histone H3 (cit-H3) (ab219407, Abcam, 1:200) antibodies were used to identify NETs in the liver.

### 2.5. Histopathological Examination of Ovarian and Liver Tissues

Ovarian and liver tissues of rats were fixed with 4% neutral paraformaldehyde buffer and embedded in paraffin. Ovary sections were stained with hematoxylin and eosin (H&E), while liver sections were stained with H&E and oil red O. Tissue morphology was evaluated under a light microscope.

### 2.6. Untargeted Metabolomics Analysis

The collected samples were thawed on ice, and metabolites were extracted with 80% methanol buffer. pooled QC samples were also prepared by combining 10 μL of each extraction mixture. All samples were acquired by the LC-MS system following machine orders. Firstly, all chromatographic separations were performed using an UltiMate 3000 UPLC System (Thermo Fisher Scientific, Bremen, Germany). An ACQUITY UPLC T3 column (100 mm × 2.1 mm, 1.8 μm, Waters, Milford, USA) was used for the reversed phase separation. The column oven was maintained at 40 °C. Then, 5 mM ammonium acetate and 5 mM acetic acid and solvent B (Acetonitrile) were added. The low rate was 0.3 mL/min and the mobile phase consisted of solvent A. Gradient elution conditions were set as follows: 0~0.8 min, 2% B; 0.8~2.8 min, 2% to 70% B; 2.8~5.6 min, 70% to 90% B; 5.6~6.4 min, 90% to 100% B; 6.4~8.0 min, 100% B; 8.0~8.1 min, 100% to 2% B; 8.1~10 min, 2%B. A high-resolution tandem mass spectrometer Q-Exactive (Thermo Scientific, Waltham, MA, USA) was used to detect metabolites eluted from the column. The Q-Exactive was operated in both positive and negative ion modes. Precursor spectra (70–1050 m/z) were collected at 70,000 resolution to hit an AGC target of 3 × 10^6^. The maximum inject time was set to 100 ms. The top 3 configurations to acquire data were set in DDA mode. Fragment spectra were collected at 17,500 resolution to hit an AGC target of 1 × 10^5^ with a maximum inject time of 80 ms. In order to evaluate the stability of the LC-MS during the whole acquisition, a quality control sample (pool of all samples) was acquired after every 10 samples.

LC−MS raw data files were converted into mzXML format and then processed by the XCMS, CAMERA, and metaX toolbox implemented with the R software (version 4.0.0). The online KEGG, HMDB database, was used to annotate the metabolites by matching the exact molecular mass data (*m*/*z*) of samples with those from the database. Statistical analysis was performed in R (version 4.0.0). Hierarchical clustering was performed using the pheatmap package. The PLSDA analysis was performed by the R package ropls and the VIP values of each variable were calculated. The three conditions of *p-*value < 0.05, difference multiple > 1.2 obtained by *t*-test, and VIP calculated by PLSDA analysis simultaneously met the screening of the final metabolites with significant differences. The software GSEA (v4.1.0) and MSigDB were used for gene set enrichment analysis to determine a set of genes in a specific KEGG pathway in different situations. Meeting the condition of |NES| > 1, NOM *p*-val < 0.05, FDR *q*-val < 0.25 was considered to be significantly different between the two groups.

### 2.7. Isolation and Culture of Mouse Bone Marrow Neutrophils

The femur and tibia of mice were separated from the surrounding tissues and washed three times with Hank’s balanced salt solution (HBSS). Bone marrow was subsequently flushed from the bones using a syringe filled with Roswell Park Memorial Institute (RPMI) medium (Gibco, Waltham, MA, USA, 11835055). Neutrophils were isolated through density gradient centrifugation using Percoll (Solarbio, Beijing, China P8370) gradients at concentrations of 55%, 65%, and 80%. The cell layers located between the 65% and 80% fractions were collected and washed with HBSS for further use. Red blood cell contamination was eliminated by lysis using a red blood cell lysis buffer (Invitrogen™, Waltham, MA, USA, 00-4300-54). Neutrophils were specifically activated to form NETs using 500 nM phorbol 12-myristate 13-acetate (PMA) (Selleck, Houston, Texas, USA, S7791), and inhibited using 10 uM GSK484 (Selleck, S7803). Cell supernatants were collected following a 4 h incubation period, and cell pellets were discarded after centrifugation at 500× *g* for 5 min. Cells were fixed with 4% paraformaldehyde and blocked using the corresponding serum. Anti-cit-H3 was used to identify NETs. Primary antibodies were incubated overnight at 4 °C, while secondary antibodies were incubated for 1 h at room temperature in the dark.

### 2.8. Cell Culture and Reagents

The mouse normal liver cell line AML12 was purchased from the Cell Bank of the Chinese Academy of Sciences (SCSP-550). AML12 cells were maintained at 37 °C in a 1:1 mixture of DMEM-F12 medium (Gibco, Waltham, MA, USA, 11330032) supplemented with 10% fetal bovine serum (Bioind), 1% insulin–transferrin–selenium (ITS) (sigma, Shanghai, China, I3146), 40 ng/mL dexamethasone, and 1% penicillin/streptomycin in a humidified incubator with 5% CO_2_. AML12 cells are commonly used to assess glycolipid metabolism in liver cells. The mouse normal liver cell line NCTC1469 was obtained from Fuheng Biology Co., LTD (Shanghai, China) (FH0334), and it was cultured at 37 °C in DMEM (Gibco, Waltham, MA, USA, 11965092) supplemented with 10% fetal bovine serum and 1% penicillin/streptomycin.

Liver cells were pretreated with various neutrophil supernatants, 500 nM PMA, or a combination of 500 nM PMA and 10 µM GSK484 for 48 h. Cells were subsequently harvested for further analysis. To assess hepatocyte alterations following insulin stimulation, the cell culture medium was replaced with a fresh medium containing 100 nM insulin and incubated for 10 min. Cells were collected for subsequent Western blot analysis. For the analysis of glucose content, insulin was incubated for a total of 4.5 h, and cell supernatants were collected at 1.5 h, 3 h, and 4.5 h intervals. Glucose content was measured according to the manufacturer’s instructions (Nanjing Jiancheng, Nanjing, China, A154-1-1) and normalized to protein concentration. To evaluate glucose uptake by hepatocytes, 100 µM 2-NBDG (MCE, New Jersey, USA, HY-116215) and insulin were co-incubated for 30 min, followed by three washes with PBS. Fluorescence intensity was measured at 485 nm (excitation) and 535 nm (emission) using an enzyme-linked immunosorbent assay reader, with normalization to protein concentration.

### 2.9. Western-Blot Analysis

Tissue homogenates and cell lysates were subjected to Western blot analysis. Protein concentrations were determined using a bicinchoninic acid (BCA) kit (Beyotime, Shanghai, China, P10010S). Proteins were separated by electrophoresis, transferred to a polyvinylidene difluoride (PVDF) membrane, and determined through chemiluminescence. The primary antibodies used included the following: anti-β-actin (4970S CST,1:1500), anti-cit-H3 (ab219407, abcam, 1:1000), anti-p-AKT (4060, CST, 1:2000), anti-AKT (4691, CST, 1:1000), anti-p-GSK (5558, CST, 1:1000), anti-GSK (5676, CST, 1:1000), anti-IRS1 (D23G12, CST, 1:1000), anti-IRS2 (4502S, CST, 1:1000), anti-PFK (89495T, CST, 1:1000), anti-PFKFB3 (13123T, CST, 1:1000), anti-PKM(4053T, CST, 1:1000), anti-GCK (sc-17819, santa cruz, 1:500), and anti-GLUT4 (66846-1-Ig, proteintech, 1:2000) antibodies. Densitometry analysis was performed using ImageJ (Version 1.52) software.

### 2.10. RNA Isolation and Real-Time PCR

Total RNA was extracted, and quantitative reverse transcription polymerase chain reaction (qRT-PCR) was performed using the ChamQ SYBR Color qPCR Master Mix (Vazyme, Shanghai, China, Q431-02) according to the manufacturer’s instructions. Primer sequences (Table 3 and Table 4).

### 2.11. Enzyme Activity

The enzymatic activities of glucokinase (GCK; SolarBio, Beijing, China, BC0745), phosphofructokinase (PFK; SolarBio, BC0535), and pyruvate kinase M (PKM; SolarBio, BC0545) in hepatic cells were quantified using commercially available kits following the manufacturer’s protocols. Briefly, cells were lysed via ultrasonication, and specific substrates were added for designated reaction periods. Absorbance at 340 nm was measured using an enzyme-linked immunosorbent assay reader, and the results were normalized to protein concentration.

### 2.12. Statistical Analysis

Statistical analyses were performed using SPSS version 26.0 (SPSS Inc., Chicago, IL, USA)and GraphPad Prism 9.0 (GraphPad Software, Inc., San Diego, CA, USA). The Shapiro–Wilk test was used to assess the normality of continuous variables. Data were presented as mean ± standard deviation (SD)/standard error of the mean (SEM) or as median (25th–75th interquartile range) for non-normally distributed variables. Student’s *t-*test and Mann–Whitney U test were employed to compare differences between the two groups. One-way analysis of variance (ANOVA) was used to compare differences among three or more independent samples, followed by post hoc pairwise comparisons with the Tukey or Bonferroni test as recommended by the software. All statistical tests were two-sided and *p*-values less than 0.05 were considered statistically significant.

## 3. Results

### 3.1. Increased Level of NETs in Serum and Follicular Fluid of PCOS Patients

**Figure 1:**
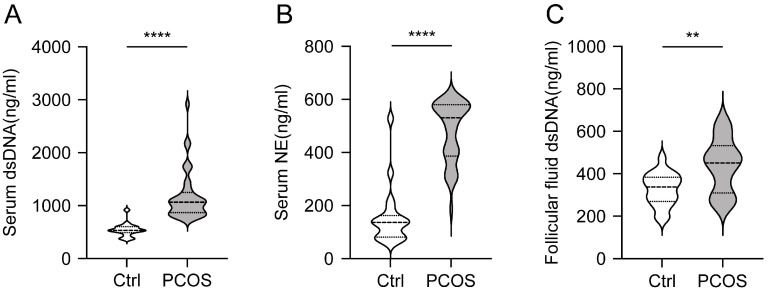
The level of NETs in serum and follicular fluid of PCOS patients. (**A**) The level of dsDNA in serum of PCOS patients. (**B**) The level of NE in serum of PCOS patients. (**C**) The level of dsDNA in follicular fluid of PCOS patients. Data are presented as mean ± SD. **, *p* < 0.01 by Student’s *t* test, ****, *p* < 0.0001 by Mann–Whitney *u* test.

To evaluate the levels of NETs in patients with PCOS, NETs markers in serum were quantified using ELISA. A cohort of 40 PCOS patients and 19 age- and BMI-matched control subjects was recruited. The results indicated that levels of dsDNA and NE were significantly higher in the PCOS group compared to the control group (*p* < 0.0001) (Figure 1A,B). Although obesity and metabolic status may influence NETs levels, we further analyzed the correlation between the clinical biochemical parameters of PCOS patients and the two NETs markers (Table S1). No significant correlation was observed between the biochemical indicators and NETs levels.

Additionally, follicular fluid samples from 17 PCOS patients and 19 age- and BMI-matched controls were analyzed. The dsDNA levels in the follicular fluid of PCOS patients were significantly higher than those in the control group (*p* = 0.0031) (Figure 1C). These findings indicate that the levels of NETs in both serum and follicular fluid are elevated in patients with PCOS.

### 3.2. Intravenous Injection of DNase I Downregulates the Formation of NETs and Ameliorates Glucose Metabolism Disorders in PCOS Rat Models

**Figure 2:**
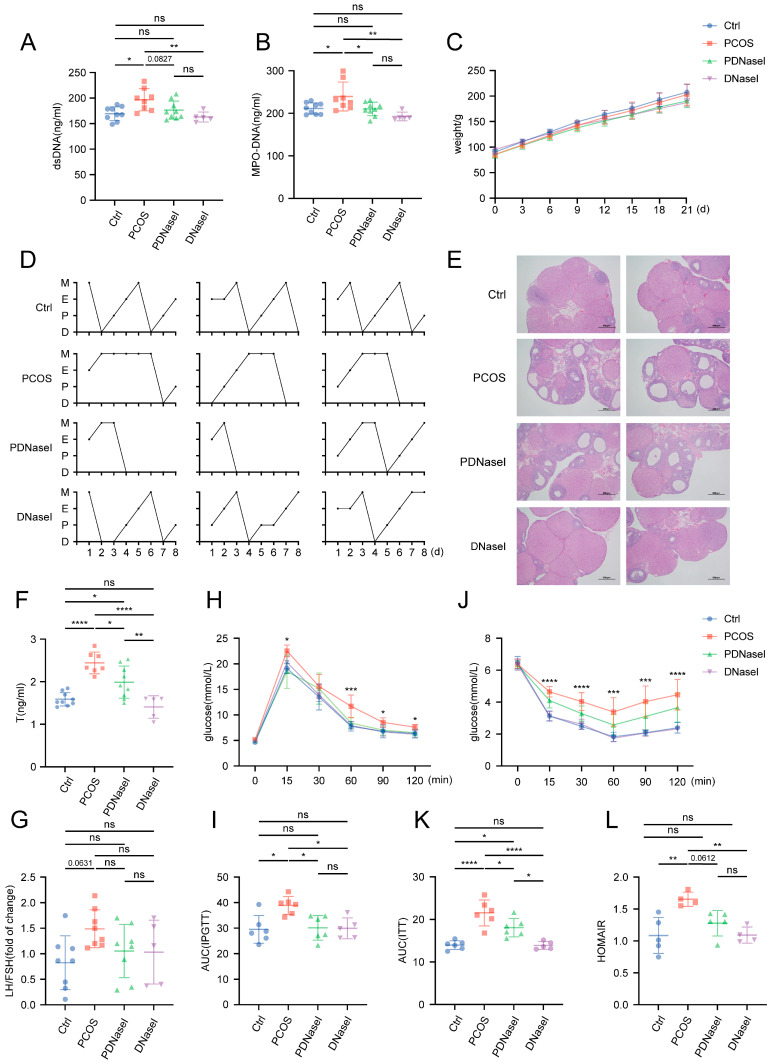
The effect of intravenous DNase I administration on DHEAs-induced PCOS rats. Serum NETs levels in the four groups of rats were assessed for (**A**) dsDNA and (**B**) MPO-DNA complex. (**C**) The growth curves of the rats’ body weight. The establishment of a DHEA-induced (60 mg/kg/day) PCOS-like model: rats were administered a therapeutic dose of DNase I via intraperitoneal injection and then evaluated for (**D**) estrous cycle analysis. D: diestrus stage; P: proestrus stage; E: estrus stage; M: metestrus stage. (**E**) H&E staining of representative ovaries showing cystic follicle or atretic follicle, antral follicle, and corpora lutea (scale bars, 500 μm). (**F**) serum testosterone and (**G**) luteinizing hormone/follicle-stimulating hormone (LH/FSH) ratios (*n* = 5–9 per group). Glucose metabolism status in rats was evaluated using (**H**) IPGTT, (**I**) AUC of IPGTT, (**J**) ITT, (**K**) AUC of ITT, and (**L**) HOMAIR. (*n* = 4–6 per group). Data are presented as mean ± SD. Data were analyzed with a one-way ANOVA test. *, *p* < 0.05, **, *p* < 0.01, ***, *p* < 0.001, ****, *p* < 0.0001. ns, no significance.

NETs are network-like structures primarily composed of dsDNA, which can be degraded by DNase I. This study aimed to investigate the potential protective effects of DNase I in mitigating elevated NETs in PCOS. A rat model of PCOS was established through subcutaneous administration of DHEAs, followed by intravenous administration of DNase I or an equivalent volume of normal saline over a 24-day period. Serum samples were collected to measure levels of dsDNA and MPO-DNA complexes, which are widely used as indicators of NETs. The PCOS group exhibited significantly elevated levels of dsDNA and MPO-DNA compared to the control group, whereas these levels were reduced in both the PDNaseI and DNaseI groups relative to the PCOS group (Figure 2A,B). These findings suggest that NETs are elevated in PCOS and that intravenous DNase I administration effectively reduces NETs formation.

The growth curves of body weight did not differ significantly among the four groups (Figure 2C). The PCOS group exhibited significant disruptions in the estrous cycle, an increased number of cystic and antral follicles, and a reduced number of corpora lutea. Similar PCOS-like characteristics were also observed in the PDNaseI group (Figure 2D,E). Analysis of sex hormone levels revealed a significant increase in T in the PCOS group, whereas the PDNaseI group demonstrated a decrease. The LH/FSH ratio was elevated in the PCOS group, with a trend towards improvement in the PDNaseI group, although the difference was not statistically significant (Figure 2F,G).

To evaluate glucose metabolism, the area under the curve (AUC) for the IPGTT and ITT was calculated. The AUC for both IPGTT and ITT was significantly increased in the PCOS group compared to the control group, indicating pronounced glucose metabolism disorders. Conversely, the AUC for both tests was significantly reduced in the PDNaseI group (Figure 2H–K). The HOMA-IR was significantly higher in the PCOS group than in the control and DNaseI groups, while the PDNaseI group exhibited a lower HOMA-IR value, although this difference did not reach statistical significance (*p* = 0.0612) (Figure 2L). Across all these indicators, the DNaseI group demonstrated similar results to the control group.

These findings indicate that the DHEAs-induced PCOS model effectively replicates the characteristics of PCOS. Intravenous administration of DNase I significantly enhances insulin sensitivity in the PCOS model, independent of obesity. However, DNase I administration does not substantially improve the disrupted estrous cycle or ovarian morphological changes.

### 3.3. DNase I May Enhance Insulin Sensitivity by Improving Liver Function

**Figure 3:**
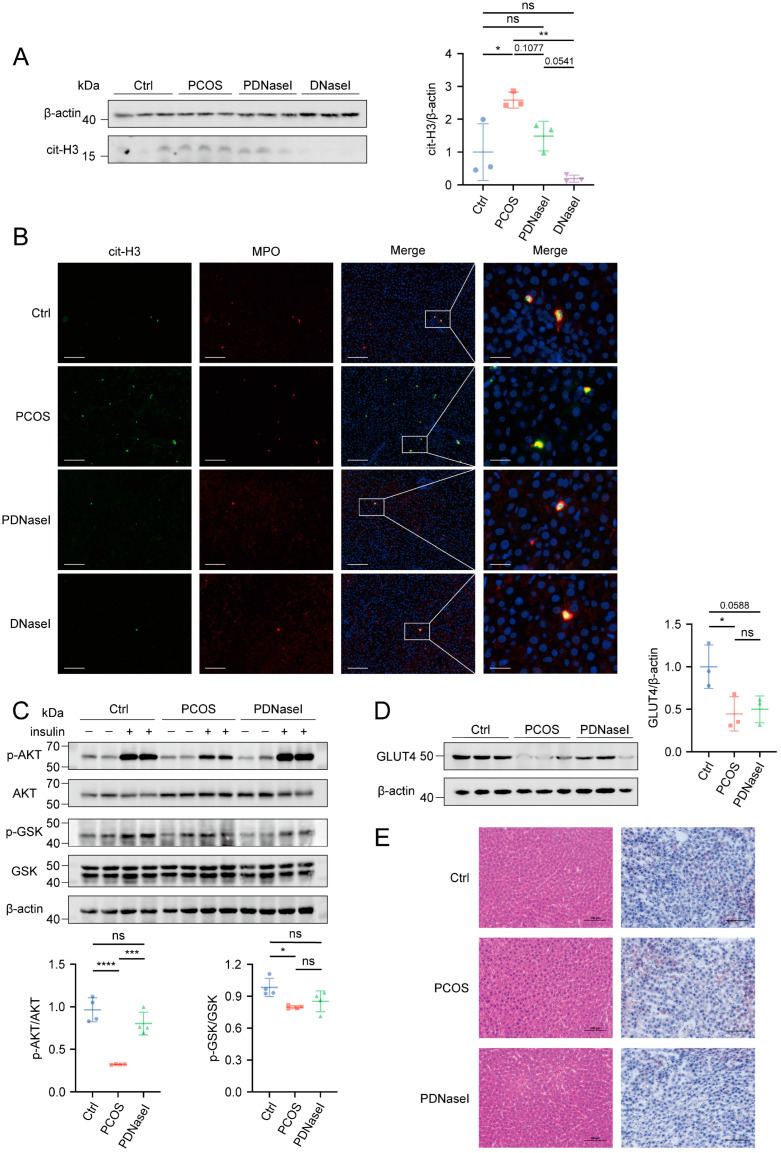
NETs were mainly concentrated in liver tissue. (**A**) NETs were quantified via histone citrullination (cit-H3) in the liver tissue (*n* = 3 per group). (**B**) Immunofluorescence analysis of co-localization of MPO and cit-H3 in liver tissue (scale bars, 100 μm, 20 μm). (**C**) Western blot analysis of p-AKT (Ser473) and p-GSKβ (Ser9) levels in livers from three groups (*n* = 2 with insulin treatment and 2 without insulin treatment). (**D**) Western blot analysis of GLUT4 levels in liver tissue from three groups (*n* = 3 per group). (**E**) Representative histology of the liver from three groups (scale bars, 100 μm). Densitometry of Western blot analysis was performed using ImageJ software. Data are presented as mean ± SD and analyzed with a one-way ANOVA test (*n* = 3–4). ns, no significance, *, *p* < 0.05, **, *p* < 0.01, ***, *p* < 0.001, ****, *p* < 0.0001.

To investigate the mechanisms underlying the significant improvement in insulin sensitivity observed in the PDNaseI group, we examined the NETs infiltration in insulin-target organs. Histone citrullination (cit-H3) levels in the liver, muscle, visceral adipose tissue (VAT), and subcutaneous adipose tissue (SAT) were quantified via Western blotting. The results revealed a significant increase in cit-H3 levels exclusively in the liver of the PCOS group compared to the control group, indicating enrichment of NETs in hepatic tissue, while no similar trends were observed in muscle or adipose tissues (Figure 3A and Figure S1A). As expected, immunofluorescence analysis confirmed the co-localization of MPO and cit-H3 in liver tissue, further supporting the presence of elevated NETs in the PCOS group (Figure 3B). Given that the DNaseI group displayed a similar trend to the control group, subsequent analyses were performed only on the control group.

We hypothesized that NETs infiltration in hepatic tissues contributes to reduced insulin sensitivity. To assess this, insulin signaling was evaluated via Western blot analysis. The results indicated a significant reduction in phosphorylated serine/threonine kinase B (p-AKT) and phosphorylated glycogen synthase kinase (p-GSK) in the liver of the PCOS group under insulin stimulation (Figure 3C). Notably, DNase I administration enhanced phosphorylation levels of insulin pathway proteins in the liver. Furthermore, we examined GLUT4 expression levels in the liver and observed a significant reduction in the PCOS group, whereas the DNaseI group exhibited levels comparable to the control group. No significant differences in GLUT4 expression were observed in other insulin-sensitive organs, including muscle, VAT, and SAT (Figure 3D and Figure S1B). Histological analysis using hematoxylin and eosin (HE) staining and oil red O staining revealed no evidence of liver fibrosis or lipid deposition in the PCOS group (Figure 3E).

These results suggest that NETs infiltration in hepatic tissue contributes to decreased insulin signal conduction in PCOS. DNase I administration ameliorates this impairment by degrading NETs in the liver.

### 3.4. NET Infiltration in the Liver Tissue Affects the Glycolytic Pathway

**Figure 4:**
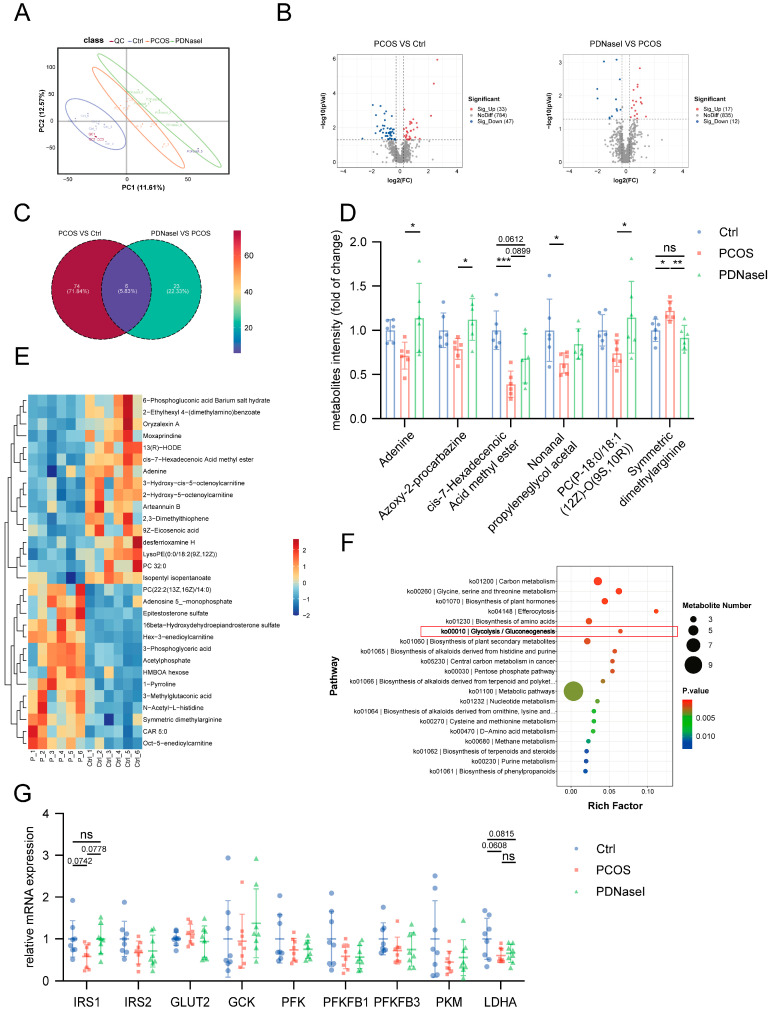
Untargeted metabolomics analyses of liver samples from three groups. (**A**) Partial least squares discriminant analysis (PLS-DA) of the metabolites across the three study groups. (**B**) Volcano plots showing the results of comparisons of metabolite levels in the PCOS group to the control group and the PDNaseI group to the PCOS group (VIP > 1, *p* < 0.05,FC > 1.2). (**C**) Venn analysis was performed for the differential metabolites of the two comparison groups. (**D**) Six metabolites common in both comparisons. (**E**) A heatmap showing the 30 top differential metabolites between the PCOS group and the control group. (**F**) KEGG pathway enrichment analysis of differential metabolites between the PCOS group and the control group. (**G**) Expression of glycolytic pathway genes of rat liver tissue among three groups. Data are presented as mean ± SD and analyzed with a one-way ANOVA test (*n* = 6–8). *, *p* < 0.05, **, *p* < 0.01, ***, *p* < 0.001. ns, no significance.

To elucidate the mechanisms underlying impaired insulin sensitivity in hepatic tissue, untargeted metabolomic analyses were performed on liver samples from the three groups (control, PCOS, PDNaseI). Partial least squares discriminant analysis (PLS-DA) revealed significant metabolic differences among the groups (Figure 4A), with an R^2^ value of 0.8449 and a Q^2^ value of −0.4104, indicating that the model was not overfitted. In the PCOS group, 33 metabolites were upregulated and 47 were downregulated compared to the control group. In the PDNaseI group, 17 metabolites were upregulated and 12 were downregulated relative to the PCOS group (Figure 4B). Among these, six metabolites were common to both comparisons (Figure 4C), specifically adenine, Azoxy-2-procarbazine, cis-7-Hexadecenoic acid methyl ester, nonanal propylene glycol acetal, PC(P-18:0/18:1(12Z)-O(9S,10R)), and symmetric dimethylarginine (Figure 4D).

Heatmapping and pathway enrichment analysis indicated significant alterations in the glycolytic pathway in the PCOS group (Figure 4E,F). To further investigate glycolytic pathway regulation, the expression levels of key glycolytic genes were examined. The results demonstrated downregulation of IRS1, IRS2, PFK, PFKFB1, PFKFB3, PKM, and LDHA in the PCOS group compared to the control group, although the differences did not reach statistical significance. (Figure 4G).

### 3.5. NETs Induce Disruptions in Glucose Utilization Within Hepatocytes

**Figure 5:**
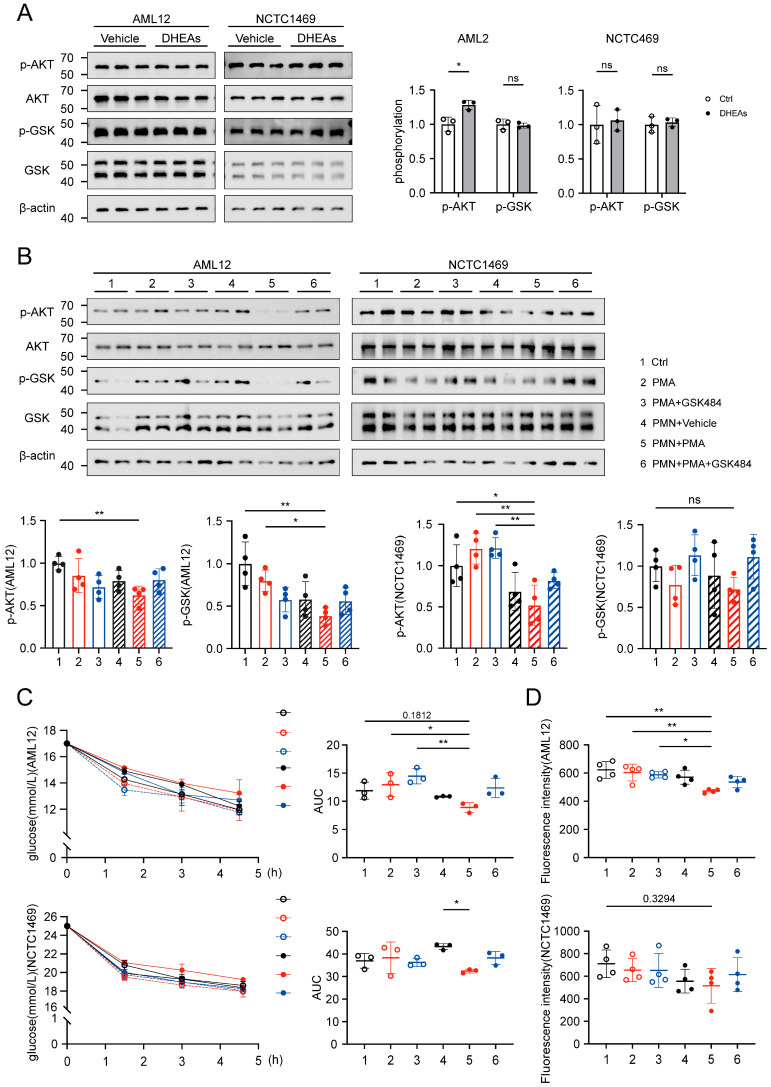
The effect of NETs on glucose utilization of hepatocytes. (**A**) Western blot analysis of p-AKT (Ser473) and p-GSKβ (Ser9) levels in AML12 and NCTC1469 treated by DHEAs (100 uM, 48 h) (*n* = 3). (**B**) Western blot analysis of p-AKT (Ser473) and p-GSKβ (Ser9) levels in AML12 and NCTC1469 cultured with neutrophil supernatant, PMA and GSK484 individually (*n* = 4). The hepatocytes were pretreated with neutrophil supernatant, PMA and GSK484 individually. A fresh medium containing 100 nM insulin was introduced and incubated. (**C**) The glucose content in the hepatocyte supernatant over 1.5, 3, and 4.5 h and AUC of the glucose consumption curve (*n* = 3). (**D**) 2-NBDG was employed as a quantitative marker. Fluorescence intensity of 2-NBDG after incubation for 30 min (*n* = 4). Data are presented as mean ± SD and analyzed with a one-way ANOVA test. *, *p* < 0.05, **, *p* < 0.01. ns, no significance.

In vitro experiments were conducted using the mouse normal liver cell lines AML12 and NCTC1469. To exclude the potential influence of DHEAs on hepatocytes, WB analysis demonstrated that DHEAs intervention did not reduce the phosphorylation levels of AKT and GSK in hepatocytes (Figure 5A), suggesting that DHEAs did not alter the phosphorylation levels of these cells. Subsequently, primary bone marrow-derived neutrophils (PMN) were isolated from mice and activated using PMA to induce NETs formation. The production of NETs was assessed through immunofluorescence, while NETs activation was inhibited using GSK484 (Supplementary Figure S1A). Neutrophil supernatants were collected and used to treat hepatocytes. Hepatocytes were cultured with neutrophil supernatant, as well as with PMA and GSK484 individually. Analysis of the insulin signaling pathway revealed that treatment with NETs-conditioned medium (CM) significantly reduced the phosphorylation of AKT and GSK (Figure 5B). Continuous monitoring of the glucose content in the hepatocyte supernatant over 1.5, 3, and 4.5 h demonstrated that glucose consumption by hepatocytes pretreated with NETs CM was significantly decreased (Figure 5C). To assess glucose uptake by hepatocytes, 2-NBDG was employed as a quantitative marker. Glucose uptake by hepatocytes treated with NETs CM was significantly reduced after 30 min (Figure 5D). Although NCTC1469 cells exhibited a downward trend in protein phosphorylation, glucose consumption, and glucose uptake, the effects were less pronounced compared to AML12 cells.

### 3.6. NETs Induce a Downregulation of the Glycolytic Pathway in Hepatocytes

**Figure 6:**
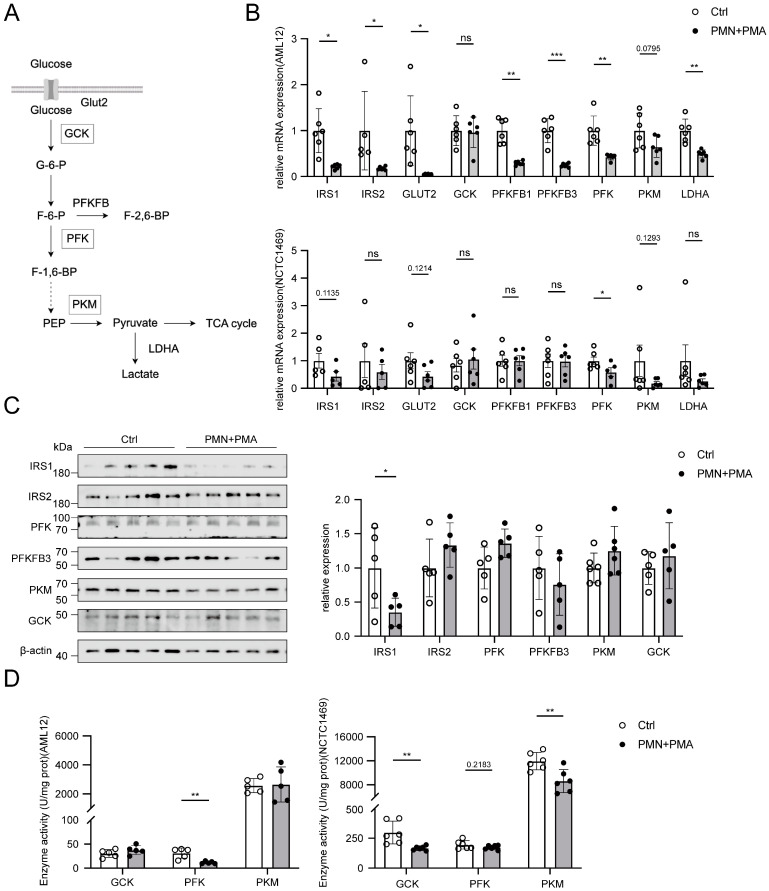
The expression of the glycolytic pathway in hepatocytes incubated with NETs CM. (**A**) Glycolytic pathway (HOME for Researchers. ID:YUYUU32283). (**B**) Expression of glycolytic pathway genes. Data are presented as mean ± SEM in NCTC1469 cells. (**C**) Expression of glycolytic pathway proteins in AML12 and (**D**) rate-limiting enzyme activities of hepatocytes treated with or without NETs CM. Data are presented as mean ± SD. Data were analyzed with Student’s *t*-test. *, *p* < 0.05, **, *p* < 0.01, ***, *p* < 0.001. ns, no significance.

Given the alterations in glycolytic pathways observed in the PCOS group through untargeted metabolomics, we assessed the expression of glycolysis-related genes and proteins in hepatocytes cultured with NETs CM (Figure 6A). qPCR analysis indicated that treatment with NETs CM resulted in a varying degree of reduction in gene expression in AML12 cells, specifically in IRS1, IRS2, GLUT2, PFKFB1, PFKFB3, PFK, and LDHA (Figure 6B). Although a downward trend was also observed in NCTC1469 cells, the changes were not statistically significant. At the protein expression level, only IRS1 was significantly downregulated in AML12 cells, while both IRS1 and PFKFB3 were significantly reduced in NCTC1469 cells. (Figure 6C and Figure S1B). Further analysis of the activity of rate-limiting enzymes in the glycolytic pathway demonstrated a significant reduction in PFK enzyme activity in AML12 cells, whereas the activities of GCK and PKM activities were significantly decreased in NCTC1469 cells (Figure 6D).

## 4. Discussion

NETs were initially identified as an immune response mechanism aimed at controlling acute bacterial infections. In recent years, extensive research has focused on the role of NETs as a contributing factor in various non-infectious diseases through multiple mechanisms, including autoimmune disorders, cardiovascular diseases, and pulmonary conditions [23]. Metabolic-related diseases, such as diabetes, nonalcoholic steatohepatitis, and obesity, have also been increasingly implicated in their pathological development. This study hypothesizes that PCOS, a reproductive endocrine disorder, is associated with elevated levels of NETs, which may impair glucose utilization by downregulating the hepatic glycolytic pathway. Inhibiting NETosis and reducing NETs-driven chronic inflammation may be involved in the improvement of insulin resistance in PCOS.

In our study, we observed a 2-3-fold increase in NETs in PCOS patients, which is consistent with the expected range reported in metabolic diseases. For example, studies in patients with diabetes have shown a 3-5-fold increase in NETs [24]. This may be due to the higher blood glucose in diabetes as neutrophils have a higher responsiveness in a high-glucose environment [20], and impaired blood glucose regulation is also one of the main characteristics of PCOS. Another meta-analysis reported that CRP in women with PCOS increased 2-fold compared with controls [25], indicating that the increase in NETs we observed is consistent with the inflammatory response in PCOS. As for NETs levels in rat modeling, we observed a tiny increase in NETs in the PCOS group. However, there have been no previous animal studies on NETs in PCOS. Another animal study of diabetes showed that NETs increased 2-3-fold in STZ-induced diabetic rats [26]. In our study, the increase in NETs found in DHEAs-induced PCOS rats was not as significant as in humans, which may be attributed to the milder degree of metabolic disorder in the DHEAs-induced modeling method.

Previous studies have documented the presence of NETs in vaginal discharge smears of PCOS patients exhibiting vaginal inflammatory responses, suggesting their involvement in the pro-inflammatory state of the vaginal microbiome in PCOS, which may adversely affect fertility in these patients [27]. In this study, we further detected elevated NETs markers in both the serum and follicular fluid of PCOS patients, suggesting that NETs are, indeed, elevated both systemically and locally. This finding implies that NETs are involved in local ovarian inflammation and may contribute to follicular development disorders in PCOS. However, in animal experiments, the removal of NETs did not ameliorate ovarian cystic follicles or estrous cycle disruptions. Regarding sex hormones, NETs removal resulted in partial improvement, probably due to improvements in metabolism. Under inflammatory conditions, liver metabolic function may be affected, altering the binding and clearance rates of sex hormones. For example, inflammation can reduce the level of sex hormone-binding globulin (SHBG) and increase the concentration of free sex hormones. We speculate that the elimination of NETs alone may be insufficient to fully restore ovarian function in PCOS. Other factors, such as granulosa cell apoptosis, endoplasmic reticulum stress, and hormonal conversion dysfunction, may play a more critical role in follicular dysplasia [28]. On the other hand, injection of DNaseI from the tail vein affects systemic circulation and may weaken local effects. If we only administer DNaseI locally to the ovaries, it may better demonstrate the role of NETs in the ovaries. Furthermore, the histological examination of ovaries should be interpreted cautiously as the rats were not euthanized at the same estrous cycle stage, which may have influenced the comparison of ovarian follicle morphology. In our study, NETs appeared to exert a greater effect on metabolic dysfunction than on reproductive abnormalities in PCOS. However, it is essential to acknowledge that while NETs significantly influence metabolic processes, they are not the sole contributing factor.

Previous research has shown that reactive oxygen species produced by neutrophils increase NETs formation in obese mice [29]. In a mouse model of metabolic dysfunction-associated steatohepatitis (MASH), the combination of alcohol and a high-fat–cholesterol–sugar diet induced hepatic stellate cells and monocytes to contribute to liver fibrosis via NETs [30]. Elevated NETs markers have also been observed in patients with nonalcoholic steatohepatitis (NASH), and the NETs inhibition has been found to modify subsequent patterns of liver inflammation, ultimately reducing hepatocellular carcinoma growth [31]. These studies highlight the presence of elevated NETs in the context of obesity and fatty liver disease. Metabolic diseases are often accompanied by chronic inflammation where oxidative stress may increase NETs formation, and inflammation may further exacerbate metabolic dysfunction, suggesting a bidirectional relationship.

Notably, the median BMI of our study population was categorized as non-obese. To mitigate the confounding effects of obesity, the PCOS cohort was matched to the control group based on BMI. Our PCOS model was established through subcutaneous administration of DHEAs. This model was characterized by reproductive anomalies, including disrupted estrous cycles, elevated serum androgen levels, increased cystic follicles, and altered glucose metabolism, without the presence of obesity, fatty liver, or compromised liver function [32,33].Consistent with these observations, all four groups of rats in our study exhibited comparable weight gain trajectories. Histological examination via HE staining and oil red O staining revealed no pathological alterations in hepatic fat deposition or fibrosis, both of which are known to influence insulin sensitivity [34]. Therefore, our findings suggest that elevated NETs in PCOS occur independently of obesity. This conclusion is consistent with results from BMI-matched PCOS patients, and the impaired insulin sensitivity observed in PCOS may be attributed to NETs rather than to lipid deposition.

In investigating the underlying mechanisms, it appears that NETs may downregulate the glycolytic pathway. We explored the changes in liver metabolic activity in three groups through untargeted metabolomics. Pathway enrichment analysis indicated significant alterations in the glycolytic pathway in the PCOS group. We did find a significant decrease in glycolysis pathway genes in liver cells treated with NETs, although we did not find significant differences in mRNA levels in liver tissue from rats, which may be due to animal heterogeneity. However, it is important to acknowledge a limitation of our study: we did not assess changes in protein levels and enzyme activity of glycolytic pathway genes in rat liver tissue. Furthermore, the six common differential metabolites of the two comparisons did not exist in the glycolysis pathway, and this aspect was not explored further. Therefore, we speculate that NETs may also affect liver metabolic function in other ways in addition to glycolysis, warranting future investigation.

Using qPCR and WB, we analyzed the expression of glycolytic pathway proteins in hepatocytes treated with NETs CM. While differences were not observed in the protein expression levels of all the glycolytic pathway genes, a significant downregulation at the mRNA level was noted for nearly all genes involved in the glycolytic pathway, albeit to varying extents. Additionally, the activity of rate-limiting enzymes was found to be significantly reduced. Consequently, we infer that the glycolytic pathway in liver cells has undergone substantial downregulation, with the decline of IRS1 and PFKFB3, potentially playing a pivotal role in this process. Previous studies have reported that neutrophil infiltration in the liver of obese mice impairs insulin signaling through elastase-mediated degradation of IRS1, a phenomenon also observed in lung cancer research [35,36]. Furthermore, decreased hepatic IRS1 levels have been noted in dihydrotestosterone (DHT)-induced PCOS rat models [37]. At the protein level, PFKFB3 exhibited a downward trend in AML12 cells, although the reduction was not statistically significant. Studies in adipocytes have demonstrated that insulin-induced phosphorylation of PFKFB3, which corresponds with glycolysis levels, acts as a positive regulator of glycolysis. Pharmacological inhibition of PFKFB3 consistently suppresses glucose uptake, GLUT4 translocation, and AKT signaling in adipocytes [38,39], which aligns with our findings.

However, it is noteworthy that upon evaluating glucose uptake and consumption along with the expression levels of glycolysis-related genes and proteins in hepatocytes treated with NETs CM, the AML12 cell line exhibited a more pronounced decrease compared to NCTC1469. We hypothesize that this disparity may be attributed to the difference in cellular characteristics as AML12 are more closely associated with hepatic glucose and lipid metabolism. Nevertheless, we acknowledge this limitation as our findings indicate that the effect of NETs on glycolysis is not consistent across all liver cell types. 

Our study provides novel evidence regarding the regulation of glucose metabolism in PCOS by NETs. However, several limitations should be acknowledged. The analysis of population sample data did not reveal a significant correlation between NETs markers and clinical indicators such as T, BMI, and HOMAIR, which may be attributable to an inadequate sample size. Furthermore, we recognize that incomplete information regarding the enrollment cohort may introduce potential bias into our findings, such as the absence of postprandial blood glucose, insulin, lipid profiles, and inflammatory cytokine data. In addition, we did not administer DNaseI after the successful modeling of PCOS induced by DHEAs, which may prevent us from completely ruling out the possibility that DNaseI reduces the efficacy of DHEAs to improve glucose metabolism disorders in PCOS rats. Additionally, the study did not explore the precise mechanisms by which the physiological environment of PCOS triggers NETs activation in the liver. This activation may result from hepatocyte-induced neutrophil chemotaxis or activation within the PCOS environment, which warrants further investigation. Based on our findings, inhibiting NETosis and reducing NETs-driven chronic inflammation may be a novel therapeutic strategy for insulin resistance intervention in PCOS.

## Figures and Tables

**Table 1:** Characteristics of serum supplied PCOS patients and controls. Data were presented as mean ± SD and median (25th–75th interquartile range) for variables with non-normal distribution.

Variables N	Ctrl 19	PCOS 40	*p*-Value
Age BMI	32.00(28.00–34.00) 21.03 ± 1.53	29.00(25.00–34.00) 21.80 ± 2.80	0.384 0.170
AMH	4.93 ± 4.08	5.37 ± 2.91	0.717
LH	5.58(4.62–7.44)	6.00(3.48–9.58)	0.536
FSH	6.58(5.04–7.17)	6.00(4.60–7.17)	0.263
LH/FSH	0.84(0.61–1.13)	1.19(0.63–1.57)	0.080
E2	42.00(32.00–64.00)	38.39(28.01–67.09)	0.729
T	0.67 ± 0.22	1.94 ± 0.94	<0.001
PRL	17.90(12.30–21.50)	10.75(8.21–17.45)	0.025
0’PG	5.05(4.73–5.38)	4.72(4.33–5.05)	0.146
0’Ins	7.05(5.70–9.31)	8.27(6.26–12.70)	0.193
HOMAIR	1.49(1.15–2.25)	1.85 (1.28–2.99)	0.472
ALT	14.00(11.00–23.00)	16.00(11.00–20.25)	0.946
AST	18.50(18.00–27.50)	17.00(15.00–19.00)	0.061

**Table 2:** Characteristics of follicular fluid supplied PCOS patients and controls. Data were presented as mean ± SD and median (25th–75th interquartile range) for variables with non-normal distribution.

Variables N	Ctrl 19	PCOS 17	*p*-Value
Age	30.56 ± 3.57	32.36 ± 3.03	0.141
BMI	22.05(19.55–25.15)	21.60(20.70–22.91)	0.812
AMH	3.91(2.46–6.66)	4.88(3.45–6.87)	0.408
LH	4.68(3.46–10.25)	5.91(4.15–8.71)	0.945
FSH	7.40 ± 3.06	6.56 ± 1.68	0.328
LH/FSH	0.95 ± 0.57	0.97 ± 0.40	0.904
E2	38.50(29.00–56.32)	33.24(29.00–52.80)	0.563
T	0.83 ± 0.34	1.79 ± 0.50	<0.001
PRL	17.60(11.58–29.38)	15.40(10.55–29.95)	0.714
0’PG	5.05 ± 0.42	5.15 ± 0.54	0.539
ALT	14.00(10.00–18.75)	15.00(11.50–18.50)	0.703
AST	16.00(14.75–21.46)	16.00(15.00–18.75)	0.754

**Table 3:** The species was a rat.

Target Gene	5′-Forward Sequences-3′	5′-Reverse Sequence-3′
IRS1	TCTACACCCGAGACGAACACT	TGGGCCCTTTGCCCGATTATG
IRS2	GACCAGTCCCACATCAGGCTT	CTGCACGGATGACCTTAGCG
GLUT2	CACCAGCACATACGACACCAGAC	CCCAAGCCACCCACCAAAGAAC
GCK	GCCGCAGTGAGGACGTGATG	AGGTGATTTCGCAGTTGGGTGTC
PFKFB1	TCAGAGCCGTACAGCCTACTACC	GCCAGAGTCACCTCCAATGCG
PFKFB3	CGGCACGGCGAGAATGAGTAC	TCAGGGCATTGGCGAACTTCTTG
PFK	TGAATCTGCGGCTGATGCTGAAG	CTCAAGGTGCGGCGTGTGAC
PKM	GTGCCGCCTGGACATTGACTC	ATTCAGCCGAGCCACATTCATCC
LDHA	GCAATCTGGATTCGGCTCGGTTC	CGGCGACATTCACACCACTCC
β-actin	GCTGTGCTATGTTGCCCTAGACTTC	GGAACCGCTCATTGCCGATAGTG

**Table 4:** The species was a mouse.

Target Gene	5′-Forward Sequences-3′	5′-Reverse Sequence-3′
IRS1	TGTCACCCAGTGGTAGTTGCTC	CTCTCAACAGGAGGTTTGGCATG
IRS2	CCAGTAAACGGAGGTGGCTACA	CCATAGACAGCTTGGAGCCACA
GLUT2	GTTGGAAGAGGAAGTCAGGGCA	ATCACGGAGACCTTCTGCTCAG
GCK	GCATCTCTGACTTCCTGGACAAG	CTTGGTCCAGTTGAGCAGGATG
PFKFB1	AGAGGCAGTGAGCTACAGGAAC	TGACCTTCCTCACGGCTGAGAT
PFKFB3	TCATCGAGTCGGTCTGTGACGA	CATGGCTTCTGCTGAGTTGCAG
PFK	CCATCAGCAACAATGTGCCTGG	TGAGGCTGACTGCTTGATGCGA
PKM	CAGAGAAGGTCTTCCTGGCTCA	GCCACATCACTGCCTTCAGCAC
LDHA	ACGCAGACAAGGAGCAGTGGAA	ATGCTCTCAGCCAAGTCTGCCA
β-actin	CATTGCTGACAGGATGCAGAAGG	TGCTGGAAGGTGGACAGTGAGG

## Data Availability

The original data presented in the study are openly available in FigShare at [DOI: 10.6084/m9.figshare.28270586].

## Supplementary Materials

The following supporting information can be downloaded at: https://www.mdpi.com/article/10.3390/biom15040572/s1, Figure S1: (A) NETs were quantified via cit-H3 in the insulin-target organs including muscle, visceral adipose tissue, and subcutaneous adipose tissue. (*n* = 4 per group). (B) Western blot analysis of GLUT4 levels in muscle, visceral adipose tissue, and subcutaneous adipose tissue. (*n* = 3 per group). (C) Representative image of neutrophil treated with PMA (500 nM) and the combination of PMA (500 nM) and GSK484 (10 μM) (scale bars, 20 μm). (D) Expression of glycolytic pathway proteins in NCTC1469. Data are presented as mean ± SD. Data were analyzed with Student’s *t*-test. *, *p* < 0.05.; Figure S2: WB images; Table S1: Correlation between serum markers of NETs and the clinical-biological parameters of PCOS subjects. Spearman’s correlation coefficient (R) and *p*-value are presented for each pair of parameters.
