# Supplementary material for: Metabolic Implications of Elevated Neutrophil Extracellular Traps in Polycystic Ovary Syndrome: A Focus on Hepatic Glycolysis
Source: Biomolecules. 2025 Apr 12;15(4):572. doi: 10.3390/biom15040572 (PMC12025135; doi:10.3390/biom15040572)

Figure 3 A

cit-H3 in liver tissues (n=3)

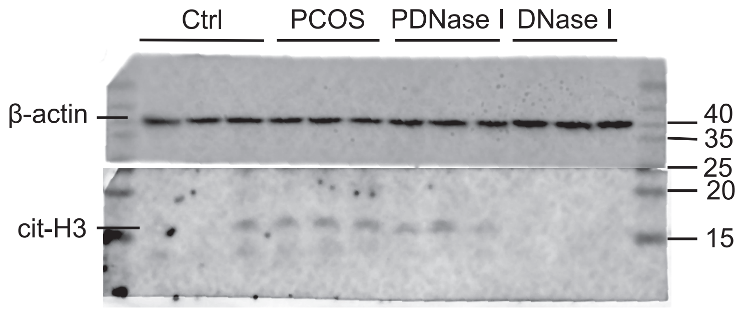

Figure 3 D

GLUT4 in liver tissues (n=3)

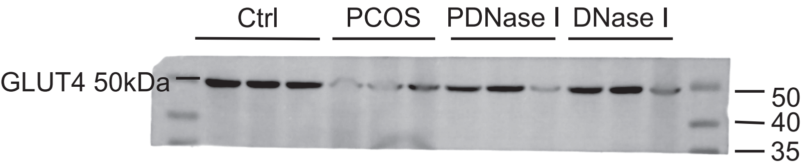

the anti-β-actin antibody was re-incubated after stripping

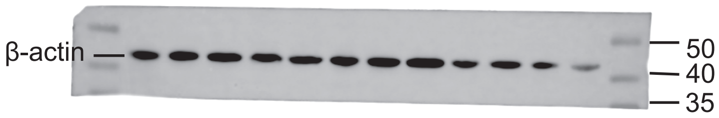

Figure S1 B

GLUT4 in muscle tissues (n=3)

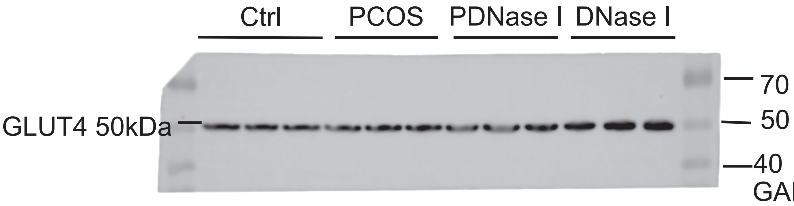

the anti-β-actin antibody was re-incubated after stripping

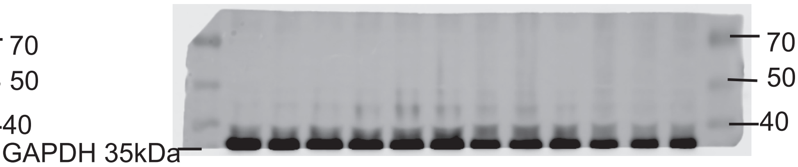

GLUT4 in VAT tissues (n=3)

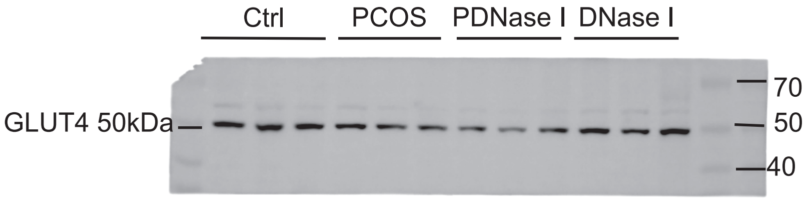

the anti-β-actin antibody was re-incubated after stripping

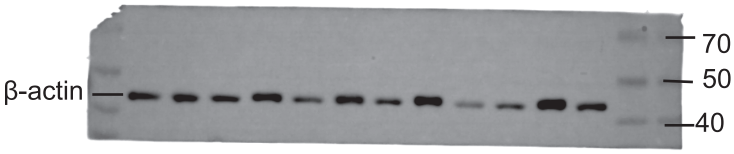

GLUT4 in SAT tissues (n=3)

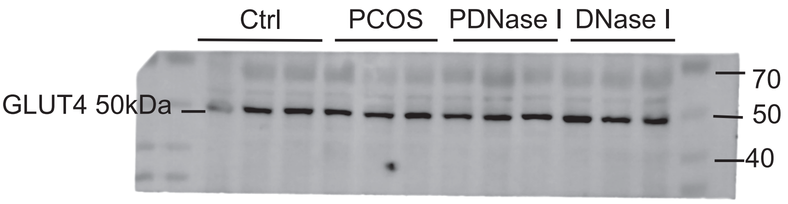

the anti-β-actin antibody was re-incubated after stripping

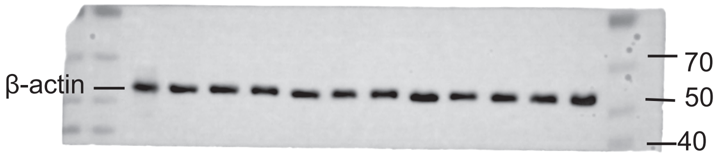

Figure 3 C

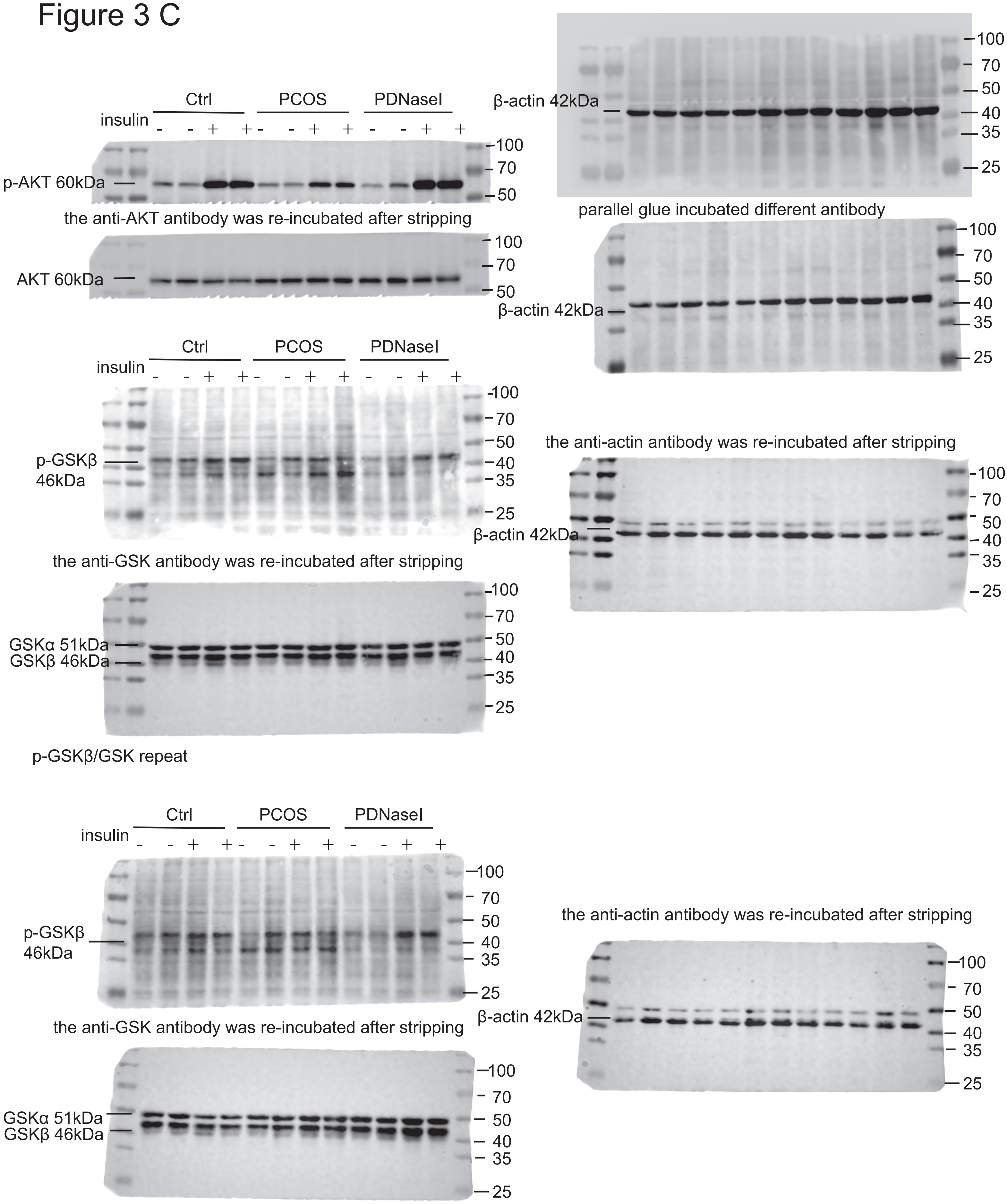
Ctrl
PCOS
PDNaseI
insulin
- - + + - - + + - - + +
p-AKT 60kDa
100
70
50
the anti-AKT antibody was re-incubated after stripping
AKT 60kDa
100
70
50
Ctrl
PCOS
PDNaseI
insulin
- - + + - - + + - - + +
p-GSKβ
46kDa
100
70
50
40
35
25
the anti-GSK antibody was re-incubated after stripping
GSKα 51kDa
GSKβ 46kDa
100
70
50
40
35
25
p-GSKβ/GSK repeat
Ctrl
PCOS
PDNaseI
insulin
- - + + - - + + - - + +
p-GSKβ
46kDa
100
70
50
40
35
25
the anti-GSK antibody was re-incubated after stripping
GSKα 51kDa
GSKβ 46kDa
100
70
50
40
35
25
β-actin 42kDa
100
70
50
40
35
25
parallel glue incubated different antibody
β-actin 42kDa
100
70
50
40
35
25
the anti-actin antibody was re-incubated after stripping
β-actin 42kDa
100
70
50
40
35
25
the anti-actin antibody was re-incubated after stripping
β-actin 42kDa
100
70
50
40
35
25

Figure 3 C

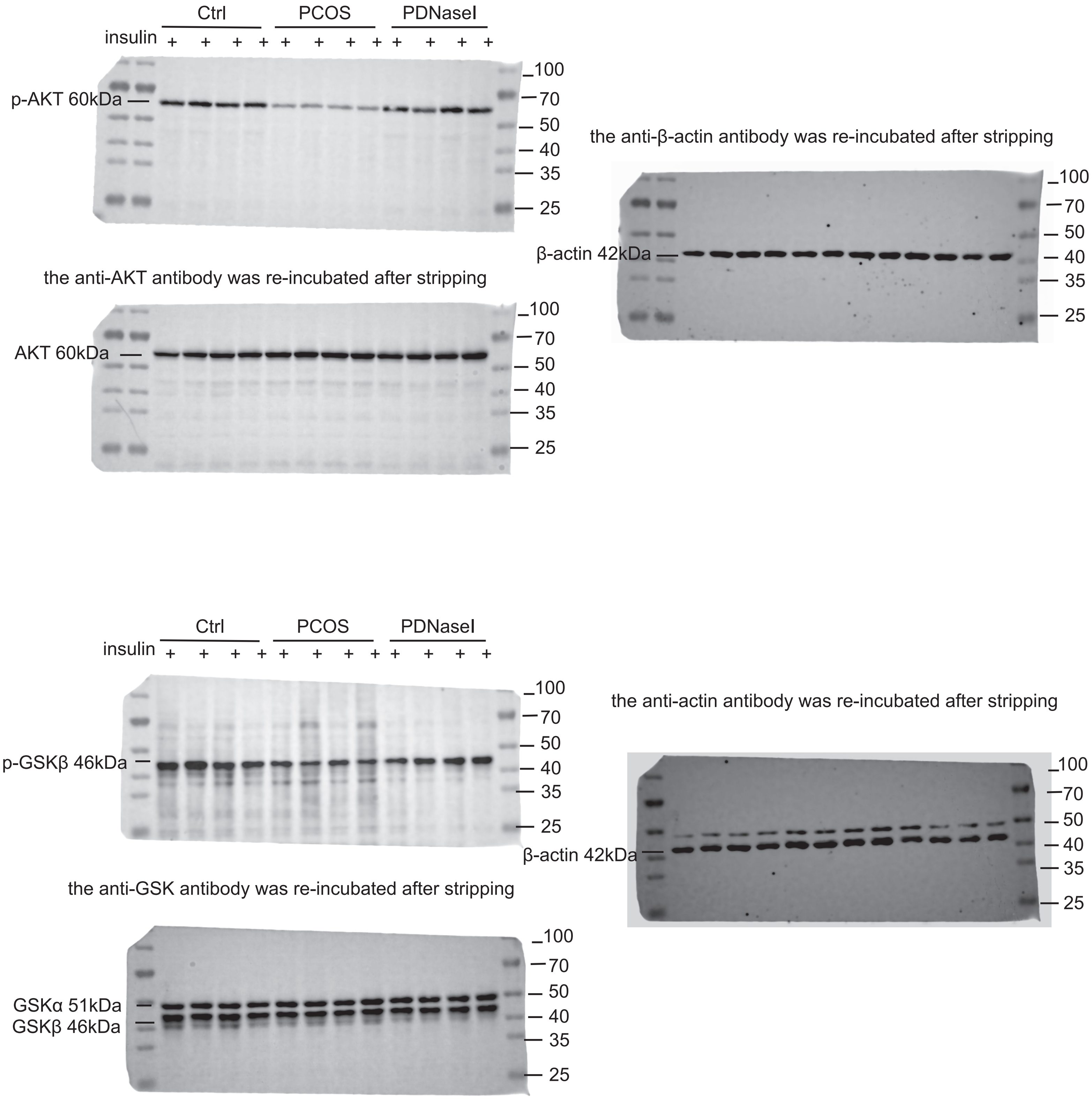
Ctrl
PCOS
PDNaseI
insulin
+ + + + + + + + + + + +
p-AKT 60kDa
100
70
50
40
35
25
the anti-AKT antibody was re-incubated after stripping
AKT 60kDa
100
70
50
40
35
25
the anti-β-actin antibody was re-incubated after stripping
β-actin 42kDa
100
70
50
40
35
25
Ctrl
PCOS
PDNaseI
insulin
+ + + + + + + + + + + +
p-GSKβ 46kDa
100
70
50
40
35
25
the anti-GSK antibody was re-incubated after stripping
GSKα 51kDa
GSKβ 46kDa
100
70
50
40
35
25
the anti-actin antibody was re-incubated after stripping
β-actin 42kDa
100
70
50
40
35
25

Figure 5 A

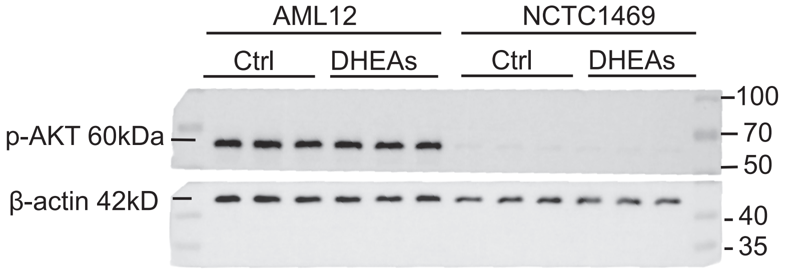

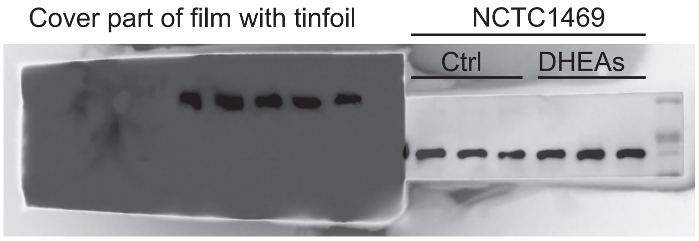

the anti-AKT antibody was re-incubated after stripping

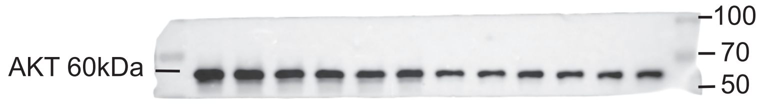

parallel glue incubated different antibody

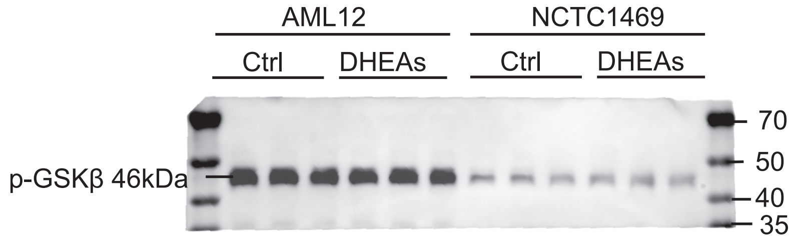

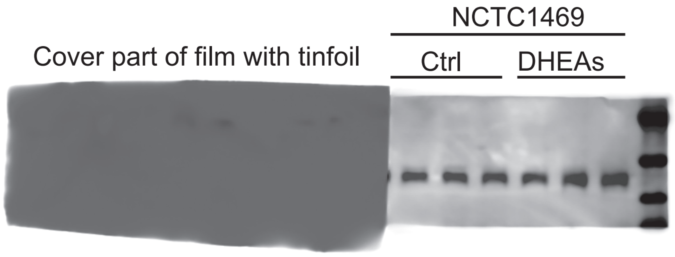

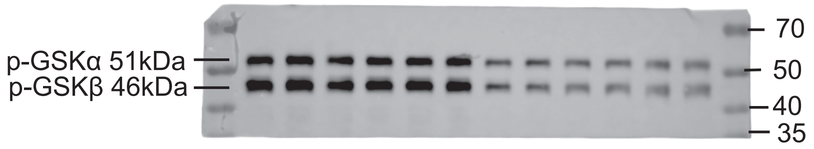

Figure 5 B AML12

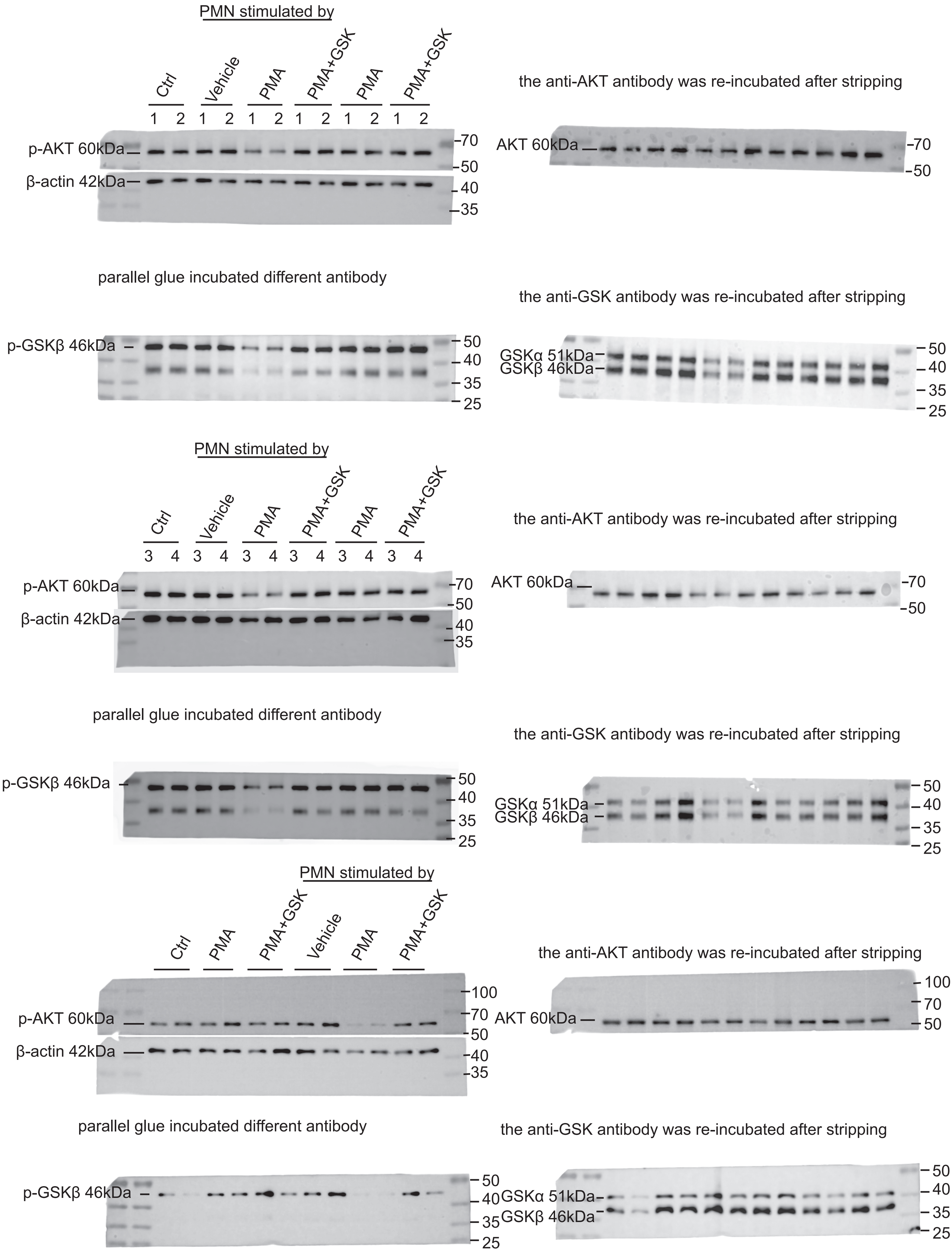

Figure 5 B NCTC1469

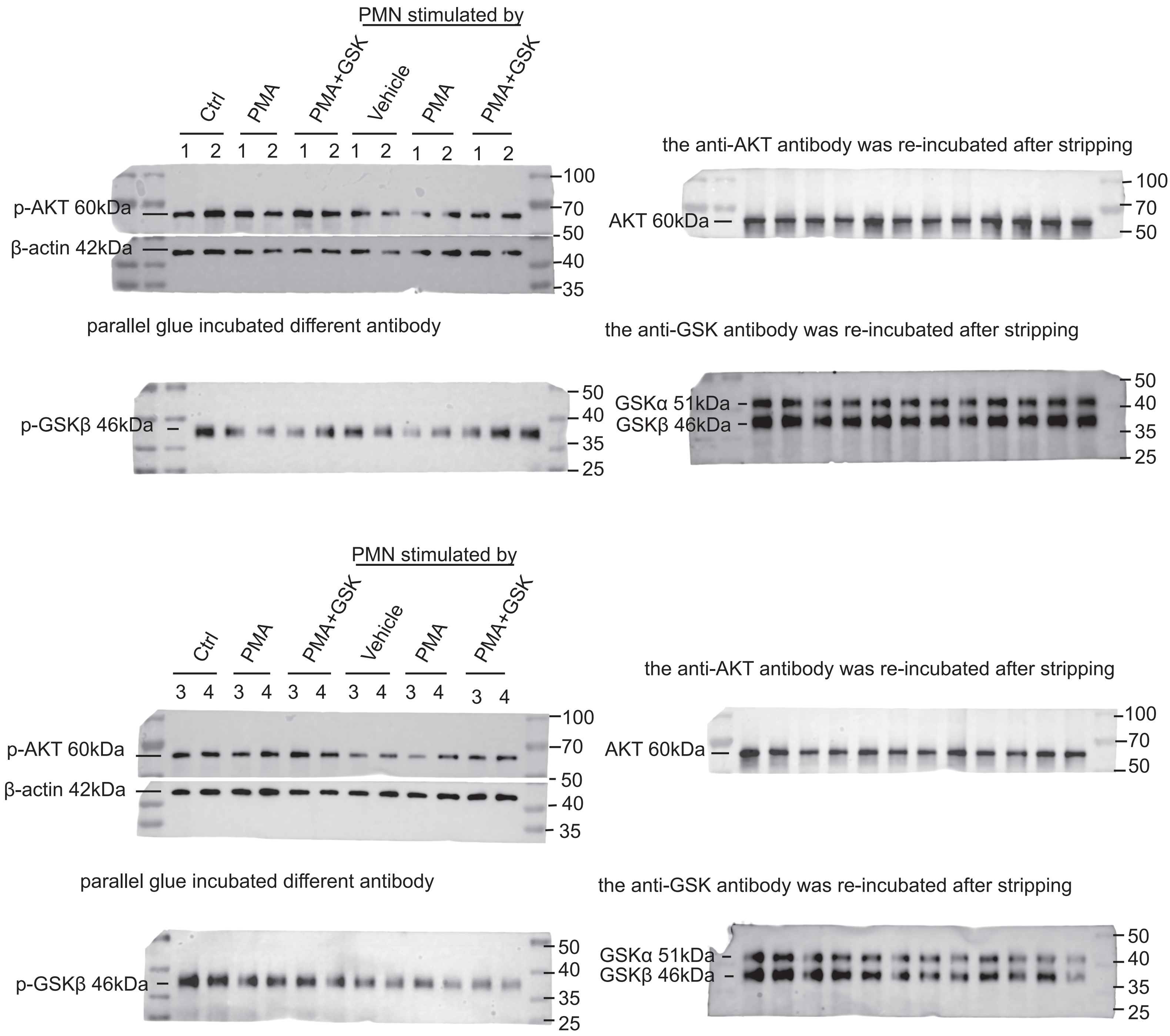

Figure 6 C AML12

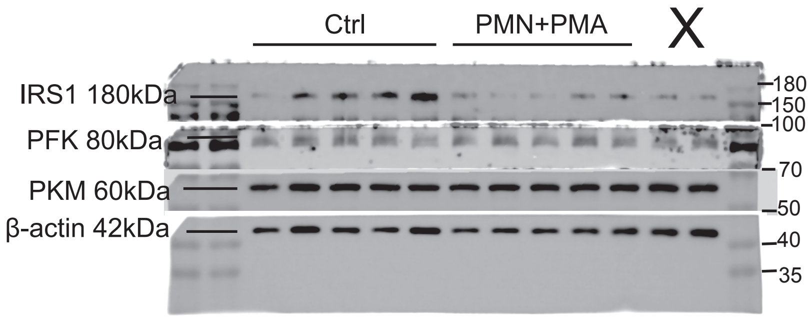

repeat

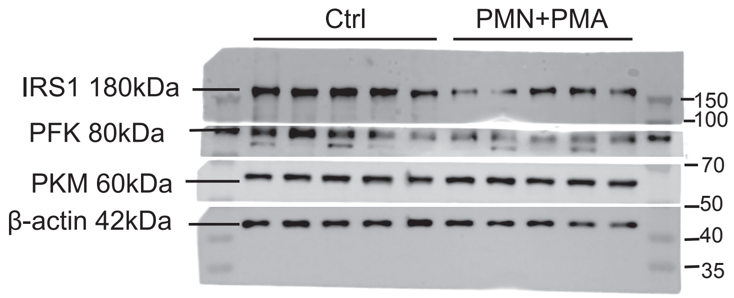

parallel glue incubated different antibody

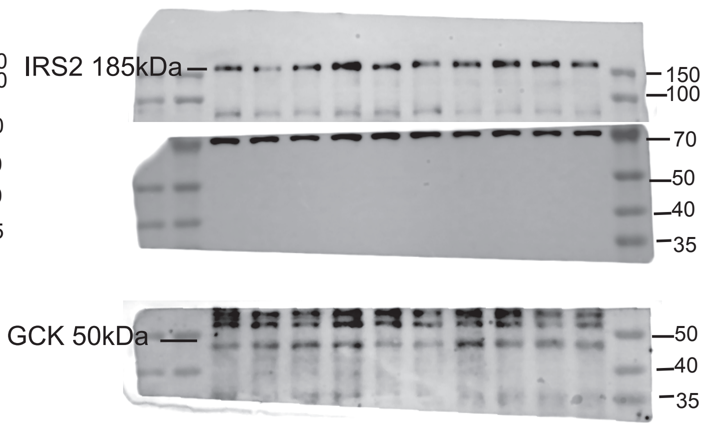

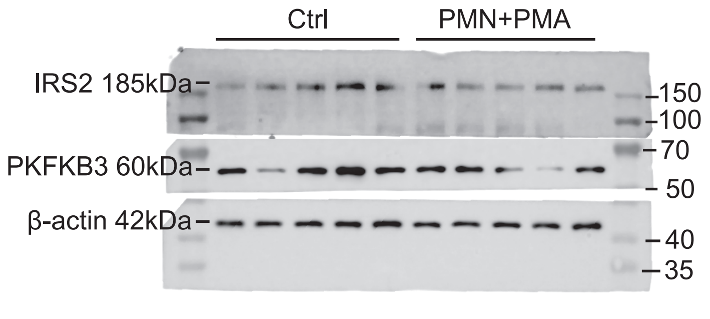

PKM repeat

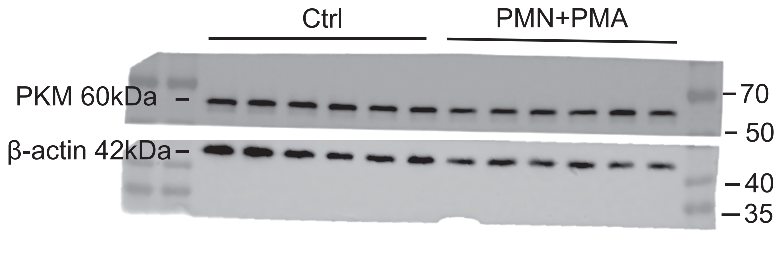

Figure S1 A

cit-H3 in liver tissues

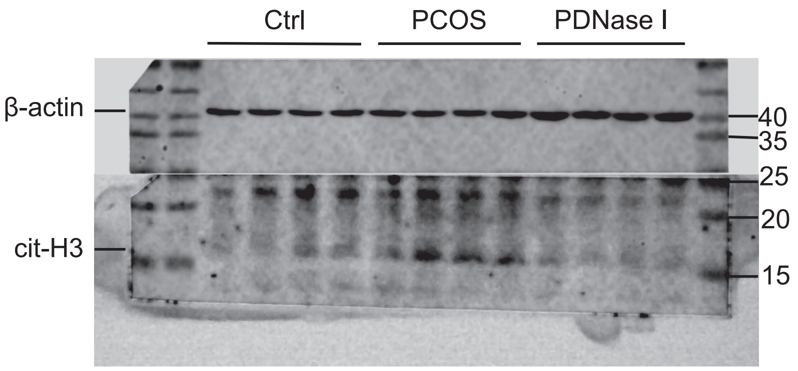

cit-H3 in liver tissues repeat

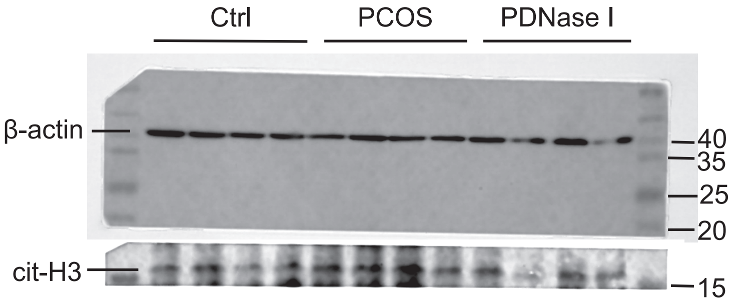

cit-H3 in muscle tissues

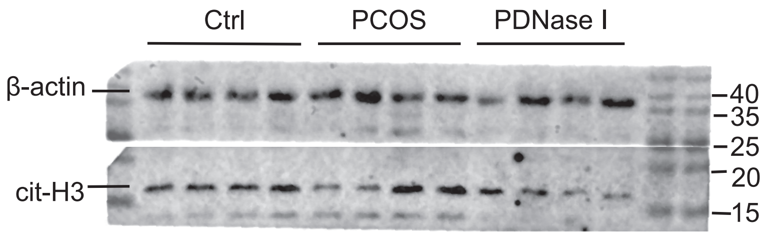

cit-H3 in VAT tissues

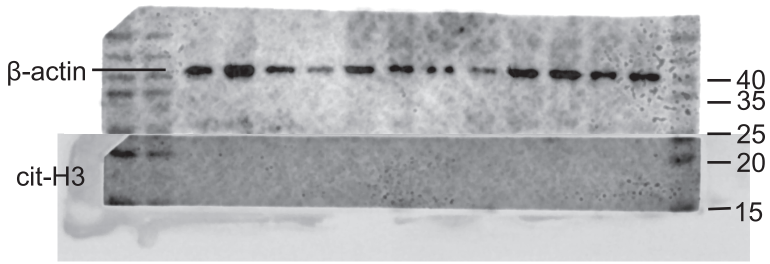

cit-H3 in SAT tissues

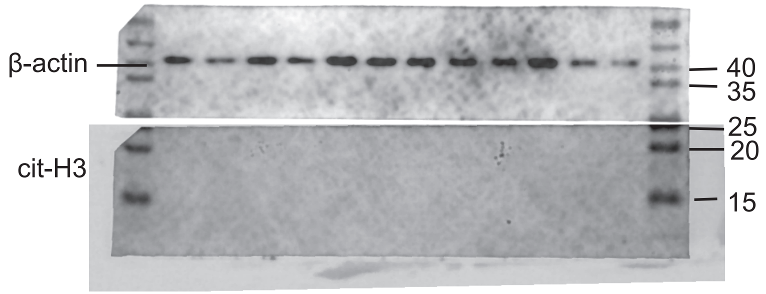

Figure S1 D NCTC1469

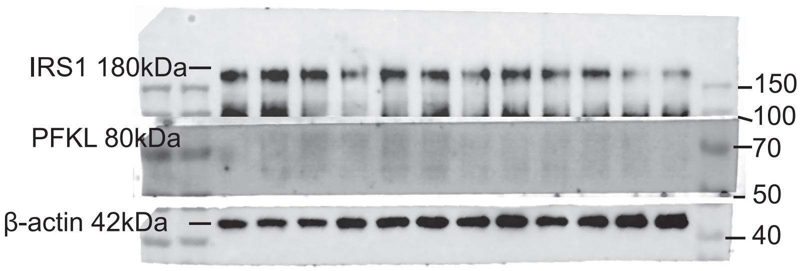

parallel glue incubated different antibody

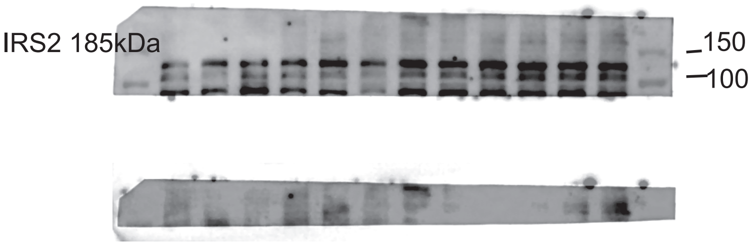

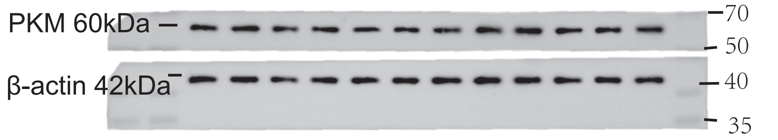

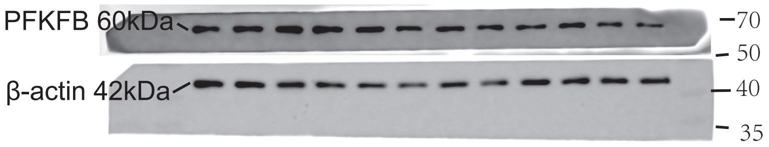

parallel glue incubated different antibody

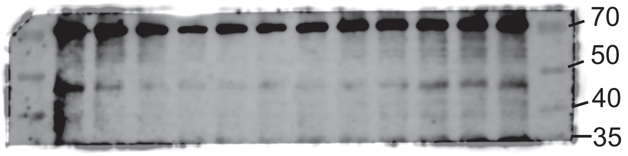

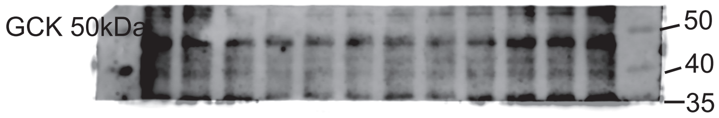

Supplement: Supplementary file 1 [file biomolecules-15-00572-s001.zip › Figure S2 WB images.pdf]
