# Supplementary material for: Metabolic Implications of Elevated Neutrophil Extracellular Traps in Polycystic Ovary Syndrome: A Focus on Hepatic Glycolysis
Source: Biomolecules. 2025 Apr 12;15(4):572. doi: 10.3390/biom15040572 (PMC12025135; doi:10.3390/biom15040572)
Supplement: Supplementary file 1 [file biomolecules-15-00572-s001.zip › Table S1.pdf]

	dsDNA		NE	
variables	R	P-value	R	P-value
BMI	0.003	0.989	-0.299	0.129
AMH	-0.023	0.909	0.071	0.767
LH	-0.115	0.540	-0.180	0.341
FSH	-0.285	0.120	-0.002	0.990
LH/FSH	0.019	0.921	-0.152	0.423
E2	-0.032	0.873	-0.318	0.106
T	0.030	0.869	0.154	0.401
PRL	-0.085	0.668	0.250	0.208
0'PG	0.220	0.173	0.038	0.819
0'Ins	0.276	0.089	-0.033	0.843
HOMA-IR	0.254	0.114	-0.055	0.740
ALT	0.071	0.701	0.287	0.118
AST	-0.139	0.473	-0.081	0.680

Table S1. Correlation between serum markers of NETs and the clinical-biological parameters of PCOS sub-jects. Spearman's correlation coefficient (R) and P-value were presented for each pair of parameters.
